# Micro-CT Assessment During Embedding of Prototype Ti Alloy Multi-Spiked Connecting Scaffold in Subchondral Trabecular Bone of Osteoarthritic Femoral Heads, Depending on Host BMI

**DOI:** 10.3390/jfb15120387

**Published:** 2024-12-21

**Authors:** Ryszard Uklejewski, Mariusz Winiecki, Adam Patalas, Patryk Mietliński, Paweł Zawadzki, Mikołaj Dąbrowski

**Affiliations:** 1 Department of Constructional Materials and Biomaterials, Faculty of Materials Engineering, Kazimierz Wielki University, Jan Karol Chodkiewicz Street 30, 85-064 Bydgoszcz, Poland; 2 Institute of Mechanical Technology, Poznan University of Technology, Piotrowo 3, 60-965 Poznan, Poland; adam.patalas@put.poznan.pl (A.P.); patryk.mietlinski@put.poznan.pl (P.M.); pawel.zawadzki@put.poznan.pl (P.Z.); 3 Adult Spine Orthopaedics Department, W. Dega Orthopaedic and Rehabilitation Clinical Hospital, Poznan University of Medical Sciences, 28 Czerwca 1956 Street 135/147, 61-545 Poznan, Poland; mdabrowski@ump.edu.pl

**Keywords:** multi-spiked connecting scaffold (MSC-Scaffold), functional biomaterial scaffold, new generation of resurfacing arthroplasty (RA) endoprosthesis, 3D-printed scaffold, micro-CT assessment, bone morphometry, osteoarthritic (OA) femoral head, body mass index (BMI), obesity

## Abstract

The prototype of a biomimetic multi-spiked connecting scaffold (MSC-Scaffold) represents an essential innovation in the fixation in subchondral trabecular bone of components for a new generation of entirely cementless hip resurfacing arthroplasty (RA) endoprostheses. In designing such a functional biomaterial scaffold, identifying the microstructural and mechanical properties of the host bone compromised by degenerative disease is crucial for proper post-operative functioning and long-term maintenance of the endoprosthesis components. This study aimed to explore, depending on the occurrence of obesity, changes in the microstructure and mechanical properties of the subchondral trabecular bone in femoral heads of osteoarthritis (OA) patients caused by the MSC-Scaffold embedding. Computed microtomography (micro-CT) scanning of femoral heads from OA patients was conducted before and after the mechanical embedding of the MSC-Scaffold. Bone morphometric parameters such as bone volume/total volume (BV/TV), trabecular thickness (Tb.Th), and trabecular number (Tb.N) for regions surrounding the MSC-Scaffold were computed, and the mechanical properties such as bone density (*ρ_B_*), bone compressive strength (*S*), and the Young’s modulus (*E*) within these regions were calculated. A statistically significant increase in BV/TV (by 15.0% and 24.9%) and Tb.Th (by 13.1% and 42.5%) and a decrease in Tb.N (by 15.2% and 23.6%) were observed, which translates to an increase in *ρ_B_* (by 15.0% and 24.9%), *S* (by 28.8% and 49.5%), and *E* (by 18.0% and 29.8%) in non-obese patients and obese patients, respectively. These changes in properties are favorable for the mechanical loads’ transfer from the artificial joint surface via the MSC-Scaffold to the periarticular trabecular bone of the OA femoral head in the postoperative period.

## 1. Introduction

The prototype multi-spiked connecting scaffold (MSC-Scaffold), manufactured using the selective laser melting (SLM) of Ti alloy powder, is an essential innovation in the fixation of components for hip resurfacing arthroplasty (RA) endoprostheses into the periarticular trabecular bone [1,2]. As a functional biomaterial, the MSC-Scaffold enables, for the first time, the biomimetic connection of RA endoprosthesis components and the uniform transfer of loads from the artificial joint surface to the periarticular trabecular bone. Its spikes replicate the natural manner of subchondral bone interlocking, which merges it with the periarticular trabecular bone to provide a gradual structural and biomechanical transition between the joint’s morphological components, such as the articular cartilage and the periarticular trabecular bone tissue of the epiphysis [3]. Such biomimetic design also facilitates the in vivo ingrowth of newly formed trabecular bone tissue within the spaces between its spikes. It reflects the natural periarticular microstructure of the joint and provides an interlocking fixation mechanism for RA endoprosthesis components. This fixation mechanism determines quite uniform stress distribution within the periarticular bone, very similar to the stress distribution in the bone of the natural hip joint [2].

During the surgical implantation of a RA endoprosthesis component with the MSC-Scaffold, its spikes are embedded in the intertrabecular space of the periarticular trabecular bone. It is therefore crucial to adjust the microgeometry of this scaffold to match the microstructure of the periarticular trabecular bone. Computed microtomography (micro-CT) assessment of animal (swine) femoral heads revealed that embedding the MSC-Scaffold prototype in the periarticular bone of femoral heads causes bone material densification under the scaffold, which in turn affects the bone’s mechanical properties in this region [4].

Hip RA endoprostheses appear to be a viable and reasonable alternative to total hip arthroplasty (THA), with a long-stem endoprosthesis for patients with osteoarthritis (OA) [5,6,7]. OA is characterized by cartilage degradation, synovial inflammation, the formation of osteophytes, and sclerosis of subchondral trabecular bone, often leading to progressive loss of function and a decline in life quality [8]. Globally, it impacts over 528 million people, or 7% of the population, including 32.5 million individuals—roughly 1 in 7 adults—in the United States (US) [9,10]. Its prevalence tends to be higher in countries with developed economies, aging populations, and elevated obesity rates [10].

Micro-CT provides high-resolution three-dimensional images that allow for detailed analysis of bone microarchitecture, including parameters such as trabecular thickness, separation, and cortical porosity [11,12]. It also enables quantitative evaluation of bone properties, such as density and stiffness, which can be correlated with mechanical properties [13]. These capabilities are critical for understanding the effects of embedding the MSC-Scaffold, as the results indicated significant bone densification, which may contribute to the scaffold’s ability to maintain its fixation in subchondral bone with initially decreased density. Moreover, micro-CT can be applied in various research areas, including osteoporosis, arthritis, and fracture healing, providing a standardized methodological approach for different bone disease models [14]. For the proper design of the MSC-Scaffold for RA endoprostheses, it is crucial to identify the microstructural and mechanical properties of the host periarticular bone of the human femoral head, which are affected by degenerative disease. These properties are important for ensuring the proper functioning in the early postoperative period and for the stability and long-term maintenance of the RA endoprosthesis components. A pilot study was conducted on a limited number of human femoral head specimens [15]. This study assessed the effect of embedding the MSC-Scaffold prototype for RA endoprostheses in the subchondral trabecular bone of OA human femoral heads using micro-CT. Its results indicated that the embedding of the MSC-Scaffold could lead to noticeable bone densification, which may contribute to the scaffold’s ability to maintain its fixation in subchondral bone with initially decreased density [15].

This study aims to assess, depending on the occurrence of obesity, changes in the microstructure and mechanical properties of the subchondral trabecular bone in human OA femoral heads (from patients treated surgically with traditional hip arthroplasty using a long-stem endoprosthesis) caused by the mechanical embedding of the MSC-Scaffold. Such studies have not been conducted so far, with the exception of the pilot study [15] carried out on a limited number of human femoral head specimens. Obtaining conclusive results from the laboratory studies on the prototype MSC-Scaffold embedding using human OA femoral heads will enable progress to the next stage of research, i.e., the experimental surgical treatment of hip OA patients using a new generation of biomimetic cementless hip RA endoprosthesis, the components of which are fixed in the periarticular bone via the MSC-Scaffold.

## 2. Materials and Methods

### 2.1. Samples of the Prototype MSC-Scaffold

The prototype MSC-Scaffold was modeled according to directives from the previous works [4,16]. In brief, the MSC-Scaffold consists of spikes shaped as truncated cones, each 5 mm in height, arranged in concentric parallel rings around a central spike, with all axes aligned parallel to one another. The central spike aligns with the axis of symmetry of the femoral head. In the MSC-Scaffold CAD model, the base diameter of each spike is 0.5 mm, and the spacing between the bases of adjacent spikes is 0.35 mm, both radially and circumferentially. Using the prepared CAD model, prototype MSC-Scaffold samples were produced via the Selective Laser Melting (SLM) technology on the REALIZER II 250 SLM^®^ machine (MTT Technologies Group, Paderborn, Germany), utilizing Ti6Al4V powder. The samples underwent specific blasting post processing to eliminate any adhered powder aggregates from the spike surfaces. Subsequently, the spike surfaces were cleaned in an ultrasonic bath (Sonic 3, Polsonic, Warszawa, Poland) using demineralized water mixed with surfactants. Details regarding the MSC-Scaffold CAD model design, the applied SLM process parameters, and the SLM post-processing treatment are given in [4].

### 2.2. Femoral Heads Specimens

Human femoral heads were acquired intraoperatively from 16 patients who underwent THA using a long-stem endoprosthesis in the W. Dega Orthopaedic and Rehabilitation Hospital of Poznan University of Medical Sciences from September 2023 to September 2024. The criteria for patients was the indication for THA treatment due to the primary OA. Basic information, such as body weight and height, was collected about the patients, which was used to determine their body mass index (BMI). Based on the BMI, the two subgroups, each consisting of 8 patients, were distinguished as non-obese patients (BMI < 30) and obese patients (BMI > 30), further referred to as NOP and OP. BMI is considered the important indicator that allows the determination of the transfer of biomechanical loads in the periarticular bone in the early postoperative period. Following surgical resection, the femoral head specimens were wrapped in dressings soaked in saline solution (NaCl 0.9%) and sealed in airtight packaging to prevent drying [17,18]. The specimens were then transported to the testing site using a portable refrigerator. Once there, they were processed under continuous water irrigation to align the femoral head-neck region and remove a portion of cartilage from the apex of the femoral head through face milling. The bone specimen preparation and preservation conditions were maintained according to the guidelines given in [19,20]; hence, for the consistency of sample quality, the total time between the femoral head specimen resection and the end of their testing was no longer than 4 h.

### 2.3. Micro-CT Assessed Embedding

#### 2.3.1. Micro-CT Scanning

The micro-CT evaluation was conducted using a GE phoenix v|tome|x s240 system (Waygate Technologies, Wunstorf, Germany). The scanning parameters included a power of 32 W (200 kV/160 µA), an exposure time of 250 milliseconds for one picture, and a voxel resolution of 14.3 µm. During each measurement, 1300 X-ray images were taken. The full scan time was approx. 17 min. The micro-CT system included the specialized executive device designed and manufactured by our research team to enable the mechanical embedding of the MSC-Scaffold in the femoral head specimens inside the micro-CT system scanning chamber [4]. Each femoral head specimen was scanned twice, before and after the mechanical embedding of the prototype Ti alloy MSC-Scaffold in the subchondral trabecular bone of OA femoral heads. The MSC-Scaffold embedding in the femoral head specimen was carried out with was carried out with a speed of 0.1 mm/s to halfway up the spikes. The embedding process was controlled radiologically to achieve the proper depth of embedding.

#### 2.3.2. Micro-CT 3D Reconstruction

3D image reconstruction of micro-CT femoral head specimens was performed by applying Phoenix datos|x 2.2 Software (Baker Hughes, Waygate Technologies, Wunstorf, Germany). Image processing included the elimination of imaging artifacts and the segmentation of images [21]. In the reconstructed bone-implant specimens, components such as the Ti-alloy MSC-Scaffold prototype, bone trabeculae, and soft tissues between the trabeculae (including bone marrow) were distinguished based on their radiological density. The same procedure was employed for identifying microstructural elements in the femoral head specimens prior to embedding the MSC-Scaffold, focusing on the two radiological phases: bone trabeculae and the soft tissues between them. Sub-resolution pores, intermediate phases, and voxels containing multiple phases were prone to misclassification [22]. To address this, a thresholding method was applied, which allocated voxels with varying grayscale values to specific phases based on predefined thresholds, thereby improving the accuracy of boundary determination for the identified microstructural elements.

#### 2.3.3. Micro-CT 2D and 3D Morphometric Measurements

For the reconstructed femoral head specimens, both before and after the mechanical embedding of the MSC-Scaffold prototype, consistent coordinate systems were established, and the datasets were analyzed using professional software (Volume Graphics 2.2, Heidelberg, Germany). Following the approach used in the pilot study [15], a cylindrical region of interest (ROI) within the femoral head reconstructions, corresponding to the MSC-Scaffold embedding site, was digitally extracted. The axis of this bone cylinder aligned with the direction of the resultant compression force exerted on the femoral head during functional leg loading in everyday physical activity [23]. The reference plane was defined as the plane tangent to the apexes of the MSC-Scaffold prototype spikes, and a series of cross-sections spaced 0.5 mm apart were used for analysis. For the cylindrical ROI in the femoral head bone prior to the MSC-Scaffold embedding, the reference plane was positioned at the same relative height with respect to the cylinder’s top. On binarized images of these cross-sections, the subchondral trabecular bone relative area was determined as the ratio of the subchondral trabecular bone area to the total area (BA/TA) using the Huang thresholding method [24]. Huang’s method is an attribute-based thresholding technique that evaluates image ‘fuzziness’, defined as the discrepancy between the grayscale and binary representations of an image [25]. This method calculates a membership function for each pixel based on its grayscale value, and the optimal threshold is determined by minimizing the fuzziness index derived from the pixel distributions of foreground and background regions [26,27]. In the context of the micro-CT analysis of subchondral trabecular bone, this approach is particularly effective in distinguishing fine structural details, where global thresholding methods may fail due to the heterogeneous intensity profiles. By leveraging object-specific attributes rather than relying solely on global intensity levels, Huang’s method ensures the precise segmentation of trabecular features, critical for accurately assessing morphometric parameters such as bone volume fraction (BV/TV) and subchondral trabecular bone area to total area (BA/TA) around the embedded MSC-Scaffold [24,28].

Based on the evaluation of change in BA/TA within the cylinder-shaped ROI of the femoral head, the spatial extent of subchondral trabecular bone densification due to the MSC-Scaffold prototype initial embedding (cf. [15]) was determined. In this area, a cuboid-shaped region of dimensions of 10 × 10 × 2.5 mm (width × depth × height) was defined. For such defined cuboid-shaped ROI, morphometric parameters such as bone volume/total volume (BV/TV), trabecular thickness (Tb.Th) and trabecular number (Tb.N) were computed. This measurement used open-source software, which was created in project R01EB021391 (Textural Biomarkers of Arthritis for the Subchondral Bone in the Temporomandibular Joint) supported by the National Institutes of Health (NIH in the USA). The input parameters, such as the mask and threshold, were deemed suitable and adequate for the hip joint.

#### 2.3.4. Mechanical Properties Calculations

The volumetric subchondral trabecular bone density *ρ_B_* was calculated using the following formula [29,30]:

*ρ_B_* = *φ*·*ρ_T_* + (1 − *φ*)·*ρ_W_* (1)

where:

*ρ_T_*—tissue mineral density of trabeculae (approximately equal to the cortical bone density, i.e., 1.85 g/cm^3^) [29,31];

*ρ_W_*—soft tissues density (approximately equal to that of water, i.e., 1.00 g/cm^3^);

*φ*—subchondral trabecular bone relative volume (BV/TV = mean BA/TA);

(1 − *φ*)—inter-trabecular soft tissue relative volume.

Then, to determine the changes in the mechanical strength of bone caused by the MSC-Scaffold embedding, the compressive strength (*S*) values of the subchondral trabecular bone in the regions of interest within the examined femoral heads, both before and after embedding, were calculated using a well-established empirical formula [32,33]:

*S* = 25·(*ρ_A_*)^1.8^, (2)

where: 

*S*—bone compressive strength [MPa]

*ρ_A_*—bone apparent density (=*φ*·*ρ_T_*) [g/cm^3^].

Young’s Modulus was calculated using the following formula [34,35]:

*E* = 7.541∙*BV*/*TV* − 0.637. (3)

This empirical formula was determined in [34] for trabecular bone of the femoral head compressed in the direction of the resultant force acting on the femoral head during everyday physical activity, which is consistent with the direction of embedding of the MSC-Scaffold.

### 2.4. Statistical Analysis

The obtained values of bone morphometric parameters—(BV/TV), (Tb.Th), and (Tb.N)—were assessed before and after the mechanical embedding of the MSC-Scaffold. To evaluate the significance of changes for each parameter before and after MSC-Scaffold embedding between the two independent subgroups (NOP and OP), statistical analysis was conducted using the paired Student *t*-test. The significance level for all the performed tests was set at *p* < 0.05. Data were processed and analyzed using Microsoft Excel software.

### 2.5. Ethics Statement

The use of femoral head samples in this research was permitted by the Bioethics Committee of the Poznan University of Medical Sciences (Poland) (Approval No. 777/2022), and all patients provided written informed consent prior to participation.

## 3. Results and Discussion

**Figure 1:**
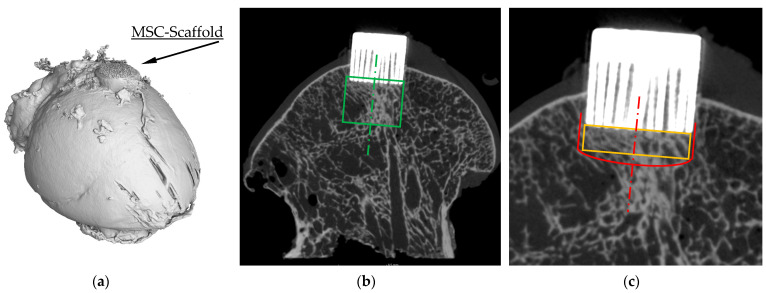
The representative (**a**) micro-CT 3D reconstruction and (**b**) cross-section of the OA femoral head specimen with the initially embedded MSC-Scaffold (cylindrical-shaped ROI marked with a green line); (**c**) the magnified fragment of the cross-section revealing the densified bone region in the peri-implant subchondral trabecular bone within which the spatial extent of subchondral trabecular bone densification due to the MSC-Scaffold prototype initial embedding (red line) and the cuboid-shaped ROI being the subject of morphometric measurements (orange line) are marked.

The representative micro-CT 3D reconstruction of the OA femoral head specimen with the initially embedded MSC-Scaffold is presented in Figure 1a, while Figure 1b presents the representative cross-section of the OA femoral head specimen with the initially embedded MSC-Scaffold with the cylindrical-shaped ROI marked. Figure 1c presents the magnified fragment of the cross-section, revealing the densified bone region in the peri-implant subchondral trabecular bone within which the spatial extent of subchondral trabecular bone densification due to the MSC-Scaffold prototype initial embedding and the cuboid-shaped ROI being the subject of morphometric measurements are marked.

**Figure 2:**
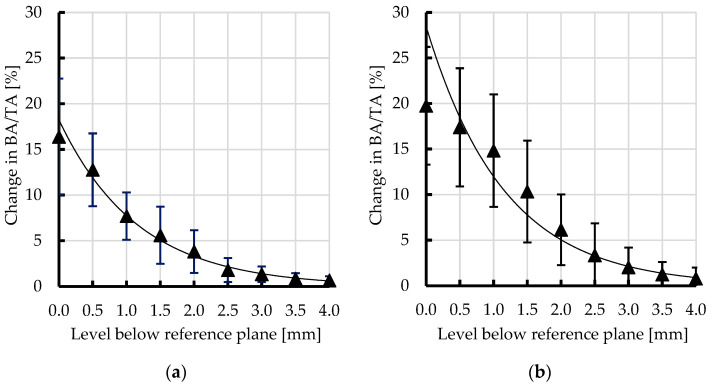
Averaged changes in subchondral trabecular bone relative area (BA/TA) values at different levels below the reference plane due to the initial embedding of the MSC-Scaffold in femoral head specimens from (**a**) NOP and (**b**) OP with OA.

The relative area values of subchondral trabecular bone (BA/TA) in femoral heads from NOP and OP with OA, as measured by micro-CT both before and after the mechanical embedding of the MSC-Scaffold, along with the percentage point changes in BA/TA values, are presented in Table 1 and Table 2, respectively. Averaged changes in BA/TA values due to the initial embedding of the MSC-Scaffold in the femoral heads for both groups are presented in Figure 2.

**Figure 3:**
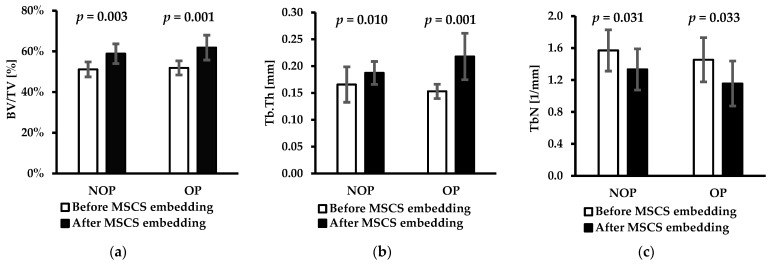
Bone morphometric parameters of subchondral trabecular bone in cuboid-shaped ROI femoral heads of OA patients before and after mechanical embedding of the MSC-Scaffold: (**a**) Bone volume/total volume (BV/TV); (**b**) Trabecular thickness (Tb.Th), and (**c**) Trabecular number (Tb.N). Data is presented as a mean ± standard deviation. Probability values (*p*-values) from the performed *t*-test between the results are given above the bars.

Results of the bone morphometry of subchondral trabecular bone in the femoral heads of OA patients before and after mechanical embedding of the MSC-Scaffold, including Bone volume/Total volume (BV/TV), Trabecular number (Tb.N), and Trabecular thickness (Tb.Tk), are presented as a mean ± standard deviation in Table 3 and Figure 3.

**Figure 4:**
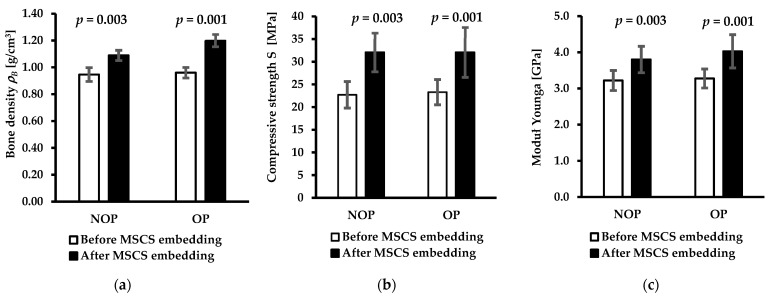
The values of the mechanical properties of subchondral trabecular bone before and after initial embedding of the MSC-Scaffold in femoral head specimens from NOP and OP with OA including (**a**) subchondral trabecular bone density *ρ_B_*, (**b**) compressive strength *S*, and (**c**) Young’s modulus *E* calculated from Formulas (1)–(3). Data are presented as a mean ± standard deviation. Probability values (*p*-values) from the performed *t*-test between the results are given above the bars.

Table 4 and Figure 4 present the data on the mechanical properties of the subchondral trabecular bone in the femoral heads of OA patients before and after mechanical embedding of the MSC-Scaffold, including the subchondral trabecular bone density *ρ_B_*, compressive strength *S*, and Young’s modulus *E*.

The BA/TA values of the subchondral trabecular bone in the femoral head specimens, as analyzed by micro-CT before the mechanical embedding of the MSC-Scaffold, showed no significant changes within the volume of each sample. The BA/TA mean values (±standard deviations) for specific femoral bone specimens varied from 50.4 ± 0.4% to 56.4 ± 0.6% for NOP and from 50.7 ± 0.9% to 55.8 ± 0.6% for OP. The BA/TA mean values for both the NOP group and OP groups are 51.4 ± 3.3 and 52.0 ± 2.3, respectively.

After the MSC-Scaffold embedding, the subchondral trabecular bone BA/TA values in micro-CT-analyzed femoral head specimens were higher, and the changes varied for particular levels below the reference plane. The BA/TA highest values were in the level growing just below the reference plane and reached a maximum value of 81.9% in the case of the femoral head specimen from the NOP group and 80.1% in the case of the femoral head specimen from the OP group. The BA/TA values gradually decreased with the distance below the reference plane to reach the same values observed before the MSC-Scaffold embedding. In the reference plane area, the mean changes in subchondral trabecular bone BA/TA were 16.4 ± 6.4% and 19.8 ± 6.5% in femoral heads collected from NOP and OP, respectively. For the levels from 0.5 mm to 2.0 mm below the reference plane range of mean changes in BA/TA, they decreased from 12.8 ± 2.1 at 0.5 mm at a distance of 0.5 mm from the reference plane to 5.6 ± 3.6 at a distance of 1.5 mm from the reference plane and from 18.9 ± 4.9 at a distance of 0.5 mm from the reference plane to 7.2 ± 3.4 at the distance of 2.0 mm from the reference plane for the femoral head specimens collected from NOP and OP, respectively. Below this level, the mean change in BA/TA values plateaued at not significant values. This allowed us to determine the spatial extent of subchondral trabecular bone densification due to the MSC-Scaffold prototype’s initial embedding. Further volumetric analyses of changes in bone morphometry and mechanical properties conducted within this area are considered significant from the point of view of changes caused by the MSC-Scaffold embedding.

From Table 3 and the diagrams presented in Figure 3 exhibiting the data on bone morphometry in the cuboid-shaped ROI, an increase in mean values of BV/TV and Tb.Th and a decrease in values of Tb.N were observed. The mean BV/TV values increased by 15.0% and 24.9% and Tb.Th by 13.1% and 42.5% for the NOP and OP, respectively, while a decrease, on average, of 15.2% and 23.6%, respectively, was observed in Tb.N values. The observed changes caused by the embedding of the MCS-Scaffold in the subchondral trabecular bone expressed by Tb.Th and Tb.N did not indicate real change in trabecular thickness and trabecular number in the measured region, but mean the changes resulting from the bone trabeculae compaction in the measured region interpreted by the software as the respectively increased trabecular thickness and decreased trabecular number. So, one can also say these measured quantities have a character of apparent quantities.

The Student *t*-statistical analysis was utilized to assess whether differences existed between the means for the groups under study are significant. A *p*-value of less than 0.031 for all the results indicated that the observed results were statistically significant at a conventional significance level (typically *p* < 0.05). This outcome implies that the measured differences can reflect the bone densification effect observed.

Based on the data collected in Table 4 and the diagrams presented in Figure 4, the improvement of mechanical properties of subchondral trabecular bone was observed due to the embedding of the MSC-Scaffold in the femoral head specimens. The relative changes in volumetric subchondral trabecular bone density *ρ_B_* with the increases of 15.0% and 24.9% for the NOP and OP groups, respectively, correspond to relative changes in BV/TV values. The compressive strength *S* mean values increased by 28.8% and 49.5%, and the Young’s modulus *E* mean values increased by 18.0% and 29.8% for the NOP and OP groups, respectively.

The MSC-Scaffold, as the original design proposed for the fixation of components of RA endoprostheses in the subchondral trabecular bone, assumes the specific method of implantation and implant fixation into the periarticular trabecular bone. This multi-spiked connecting scaffold is a functional biomaterial, enabling, for the first time, the biomimetic connection and fixation of RA endoprostheses components and the uniform transfer of loads from the artificial joint surface to the periarticular bone. The primary fixation of the RA endoprostheses components is assumed to consist of two stages. The fixation first stage involves the surgical implantation of the RA endoprosthesis components into the peri-articular trabecular bone, reaching about half the height of the MSC-Scaffold spikes, performed by the operating surgeon. In contrast, the second stage is the biological fixation, which occurs as bone tissue grows into the remaining inter-spike regions of the MSC-Scaffold during the post-operative rehabilitation of OA patients.

The bone damage and compaction due to the MSC-Scaffold embedding is a kind of functional densification of the bone tissue, which should ensure proper functioning in the early postoperative period. It is possible that with healing after the surgery, it will result in the repair of the trabecular fractures and overall consolidation of bone tissue to achieve final biological fixation and stability of the RA endoprosthesis.

The mechanical quality of the bone stock of patients qualified for surgery with RA endoprostheses is generally weakened due to OA. In the early postoperative period, no bone ingrowth is yet occurring or it is in its initial phase. For this reason crucial is a realistic evaluation of the subchondral trabecular bone condition of such patients. It is important for the successful postoperative functioning of the RA endoprosthesis with the MSC-Scaffold to ensure the load transfer in the trabecular bone within the range of its elastic deformations, which would prevent further implant embedding and its migration. It is of critical importance for the further stability and maintenance of the RA endoprosthesis components.

The results obtained in this study on human OA femoral heads confirm the working hypotheses from the previous pilot study [15]—the densification of the subchondral trabecular bone occurring as a result of mechanical embedding of the MSC-Scaffold improves the mechanical properties of the trabecular bone under the scaffold (bone density *ρ_B_* by 15.0% and 24.9%, compressive strength *S* by 28.8% and 49.5%, and Young’s modulus *E* by 18.0% and 29.8%, for the NOP and OP groups, respectively).

The conducted studies on the morphometry and mechanical properties of the OA bone in the scaffold surroundings and the changes in this region as a result of the MSC-Scaffold embedding will allow the use of the obtained results to conduct realistic biomechanical numerical simulations to design the appropriate geometry of the MSC-Scaffold, enabling the transfer of loads from the scaffold to the bone within a safe range of elastic deformations. MSC-Scaffold studies on human femoral heads with OA will allow us to proceed to the next step, the surgical stage of research, i.e., experimental surgical treatment using a prototype RA endoprosthesis with the MSC-Scaffold.

## 4. Conclusions

The embedding of the prototype Ti alloy MSC-Scaffold in the femoral head causes trabecular compaction in the region under the spikes of the MSC-Scaffold and an increase in the density of the subchondral trabecular bone of the OA femoral head. The spatial extent of subchondral trabecular bone densification due to the MSC-Scaffold initial embedding ranges from 1.5 to 2.5 mm, which is about half the height of the MSC-Scaffold’s spikes when embedded up to half the height of the spikes.

The densification of the subchondral trabecular bone in the region under the scaffold identified by the statistically significant increase in BV/TV (by 15.0% and 24.9%) and Tb.Th (by 13.1% and 42.5%) and decrease in Tb.N (15.2% and 23.6%) values results in the increase in the values of mechanical parameters of bone for the NOP and OP groups, respectively. It was stated that the statistically significant increases in the compressive strength *S* of subchondral trabecular bone averaged 28.8% and 49.5%, and the Young’s modulus *E* averaged 18.0% and 29.8% for the NOP and OP groups, respectively.

The observed changes in the values of the mechanical parameters of the bone in the region under the embedded scaffold are favorable for the conditions of transferring mechanical loads to the OA bone head in the postoperative period. The increased strength of the subchondral trabecular bone can counteract the possible migration of the implant during the postoperative limb loading.

## Figures and Tables

**Table 1:** Subchondral trabecular bone relative area (BA/TA) values in femoral heads from NOP with OA measured in micro-CT before and after mechanical embedding of the MSC-Scaffold (MSCS) and the percentage point changes in BA/TA values; * spatial extent of subchondral trabecular bone densification due to the MSC-Scaffold prototype initial embedding [mm].

Patient No.		Levels Below Reference Plane [mm]									Mean ± SD
		0.0	0.5	1.0	1.5	2.0	2.5	3.0	3.5	4.0	
1	BA/TA before MSCS embedding [%]	57.6	56.9	56.2	56.8	55.8	55.9	56.1	55.8	56.4	56.4 ± 0.6
	BA/TA after MSCS embedding [%]	74.4	69.5	63.4	59.8	57.2	56.8	56.9	57.3	57.1	
	Change in BA/TA [%]	16.8	12.6	7.2	3.0 *	1.4	0.9	0.8	1.5	0.7	
2	BA/TA before MSCS embedding [%]	52.1	51.1	51.8	52.8	51.6	52.4	51.1	51.3	52	51.8 ± 0.6
	BA/TA after MSCS embedding [%]	68.9	67.1	61.2	59.7	56.1	53.4	52.7	52.2	52.8	
	Change in BA/TA [%]	16.8	16.0	9.4	6.9	4.5 *	1.0	1.6	0.9	0.8	
3	BA/TA before MSCS embedding [%]	51.2	50.4	50.3	50.6	49.9	49.8	50.1	50.4	50.8	50.4 ± 0.4
	BA/TA after MSCS embedding [%]	68.3	65.4	62.1	60.1	57.3	53.4	52.4	51.8	51.9	
	Change in BA/TA [%]	17.1	15.0	11.8	9.5	7.4	3.6 *	2.3	1.4	1.1	
4	BA/TA before MSCS embedding [%]	56.2	56.3	55.5	55.9	55.1	54.2	54.8	54.9	54.9	55.4 ± 0.7
	BA/TA after MSCS embedding [%]	68.2	67.1	59.2	58.3	57.4	57.8	57.1	56.6	56.4	
	Change in BA/TA [%]	12.0	10.8	3.7	2.4	2.3	3.6 *	2.3	1.7	1.5	
5	BA/TA before MSCS embedding [%]	53.4	52.7	53.4	51.1	50.9	52.4	52.8	52.3	52.4	52.4 ± 0.9
	BA/TA after MSCS embedding [%]	81.9	72.1	58.9	57.7	54.5	52.8	53.2	52.6	52.9	
	Change in BA/TA [%]	28.5	19.4	5.5	6.6	3.6 *	0.4	0.4	0.3	0.5	
6	BA/TA before MSCS embedding [%]	46.2	46.8	45.2	44.8	44.6	44.8	45.3	46.7	45.7	45.6 ± 0.8
	BA/TA after MSCS embedding [%]	58.2	56.1	52.6	46.1	45.8	46.2	45.9	46.8	45.9	
	Change in BA/TA [%]	12.0	9.3	7.4 *	1.3	1.2	1.4	0.6	0.1	0.2	
7	BA/TA before MSCS embedding [%]	48.7	48.6	47.9	48.1	48.3	49.6	49.7	50.2	49.8	49.0 ± 0.8
	BA/TA after MSCS embedding [%]	69.2	60.8	57.9	57.6	55.2	52.4	51.9	50.7	49.3	
	Change in BA/TA [%]	20.5	12.2	10.0	9.5	6.9 *	2.8	2.2	0.5	0.2	
8	BA/TA before MSCS embedding [%]	52.1	51.3	50.2	49.6	50.3	51.1	50.8	50.1	50.7	50.7 ± 0.7
	BA/TA after MSCS embedding [%]	59.5	58.1	56.8	55.2	53.6	51.8	51.3	50.5	50.9	
	Change in BA/TA [%]	7.4	6.8	6.6	5.6	3.3 *	0.7	0.5	0.4	0.2	
	Mean ± SD of BA/TA before MSCS embedding [%]	-	-	-	-	-	-	-	-	-	51.4 ± 3.3
	Mean ± SD of change of BA/TA [%]	16.4 ± 6.4	12.8 ± 4.0	7.7 ± 2.6	5.6 ± 3.1	3.8 ± 2.3 *	1.8 ± 1.3	1.3 ± 0.9	0.9 ± 0.6	0.6 ± 0.5	-

**Table 2:** Subchondral trabecular bone relative area (BA/TA) values in femoral heads from OP with OA measured in micro-CT before and after embedding of the MSC-Scaffold (MSCS) and the percentage point changes in BA/TA values; * spatial extent of subchondral trabecular bone densification due to the MSC-Scaffold prototype initial embedding [mm].

Patient No.		Levels Below Reference Plane [mm]									Mean ± SD
		0.0	0.5	1.0	1.5	2.0	2.5	3.0	3.5	4.0	
1	BA/TA before MSCS embedding [%]	52.3	51.6	49.2	51.1	52.8	53.6	54.1	52.4	52.7	52.2 ± 1.4
	BA/TA after MSCS embedding [%]	78.8	73.8	72.6	68.1	57.7	56.8	55.2	54.8	54.2	
	Change in BA/TA [%]	26.5	22.2	23.4	17.0	4.9	3.2 *	1.1	2.4	1.5	
2	BA/TA before MSCS embedding [%]	56.4	55.1	55.2	56.0	56.7	56.4	55.1	55.7	55.9	55.8 ± 0.6
	BA/TA after MSCS embedding [%]	78.2	74.6	71.4	65.2	62.4	58.7	57.1	56.8	56.0	
	Change in BA/TA [%]	21.8	19.5	16.2	9.2	5.7 *	2.3	2.0	1.1	0.1	
3	BA/TA before MSCS embedding [%]	51.2	52.1	51.6	51.3	50.4	50.1	49.9	49.7	49.6	50.7 ± 0.9
	BA/TA after MSCS embedding [%]	80.1	79.3	73.6	70.4	62.4	57.3	53.4	53.6	53.1	
	Change in BA/TA [%]	28.9	27.2	22.0	19.1	12.0	7.2	3.5	3.9	3.5	
4	BA/TA before MSCS embedding [%]	55.5	55.1	54.6	54.9	55.7	55.2	54.6	55.6	55.1	55.1 ± 0.4
	BA/TA after MSCS embedding [%]	78.6	77.7	73.1	64.9	61.4	55.2	55.2	55.7	55,2	
	Change in BA/TA [%]	23.1	22.6	18.5	10.0	5.7 *	0.0	0.6	0.1	0.1	
5	BA/TA before MSCS embedding [%]	51.8	52.3	51.4	50.6	50.4	49.8	50.2	50.6	49.6	50.7 ± 0.9
	BA/TA after MSCS embedding [%]	66.4	65.1	60.4	60.2	58.2	54.3	51.8	51.2	50.2	
	Change in BA/TA [%]	14.6	12.8	9.0	9.6	7.8	4.5 *	1.6	0.6	0.6	
6	BA/TA before MSCS embedding [%]	48.3	49.6	49.9	50.1	50.6	50.2	48.6	48.2	47.9	49.3 ± 1.0
	BA/TA after MSCS embedding [%]	67.4	64.7	63.1	60.4	61.1	59.6	55.3	49.8	48.2	
	Change in BA/TA [%]	19.1	15.1	13.2	10.3	10.5	9.4	6.7 *	1.6	0.3	
7	BA/TA before MSCS embedding [%]	51.6	52.1	51.6	51.2	50.8	51.7	51.3	51.4	50.9	51.4 ± 0.4
	BA/TA after MSCS embedding [%]	63.4	63.3	60.2	52.9	52.1	51.8	51.5	51.5	51.1	
	Change in BA/TA [%]	11.8	11.2	8.6 *	1.7	1.3	0.1	0.6	0.1	0.2	
8	BA/TA before MSCS embedding [%]	51.1	50.9	50.1	50.6	50.7	51.2	50.8	49.9	49.9	50.6 ± 0.5
	BA/TA after MSCS embedding [%]	63.3	59.4	57.8	56.4	51.9	51.2	50.8	49.6	49.9	
	Change in BA/TA [%]	12.2	8.5	7.7	5.8 *	1.2	0.0	0.0	0.0	0.0	
	Mean ± SD of BA/TA before MSCS embedding [%]	-	-	-	-	-	-	-	-	-	52.0 ± 2.3
	Mean ± SD of change of BA/TA [%]	19.8 ± 6.5	17.4 ± 6.5	14.8 ± 6.2	10.3 ± 5.6	6.1 ± 3.9	3.3 ± 3.5*	2.0 ± 2.2	1.2 ± 1.4	0.8 ± 1.2	-

**Table 3:** Bone morphometric parameters of subchondral trabecular bone in cuboid-shaped ROI of femoral head specimens from NOP and OP patients with OA before and after embedding of the MSC-Scaffold (MSCS) in femoral head specimens from NOP and OP with OA. ↑ and ↓ indicate an increase or decrease of the Relative Change [%], respectively.

Parameter	NOP				OP			
	Before MSCS Embedding	After MSCS Embedding	Relative Change [%]	Statistical Significance *p*-Values	Before MSCS Embedding	After MSCS Embedding	Relative Change [%]	Statistical Significance *p*-Values
BV/TV [%]	51.2 ± 3.7	58.9 ± 4.8	↑15.0	0.003	51.9 ± 3.5	64.8 ± 5.4	↑24.9	0.001
Tb.Th [mm]	0.166 ± 0.033	0.187 ± 0.021	↑13.1	0.010	0.153 ± 0.013	0.201 ± 0.043	↑42.5	0.001
Tb.N [1/mm]	1.57 ± 0.26	1.33 ± 0.26	↓15.2	0.031	1.45 ± 0.28	1.11 ± 0.28	↓23.6	0.033

**Table 4:** The values of mechanical properties of subchondral trabecular bone before and after initial embedding of the MSC-Scaffold (MSCS) in femoral head specimens from NOP and OP with OA, including subchondral trabecular bone density *ρ_B_*, compressive strength *S*, and Young’s modulus *E* calculated from Formulas (1)–(3). ↑ and ↓ indicate an increase or decrease of the Relative Change [%], respectively.

Parameter	NOP				OP			
	Before MSCS Embedding	After MSCS Embedding	Relative Change [%]	Statistical Significance *p*-Values	Before MSCS Embedding	After MSCS Embedding	Relative Change [%]	Statistical Significance *p*-Values
*ρ_B_* [g/cm^3^]	0.95 ± 0.07	1.09 ± 0.09	↑15.0	0.003	0.96 ± 0.06	1.20 ± 0.10	↑24.9	0.001
*S* [MPa]	22.7 ± 2.9	29.3 ± 3.43	↑28.8	0.003	23.3 ± 2.9	34.8 ± 5.2	↑49.5	0.001
*E* [GPa]	3.22 ± 0.28	3.80 ± 0.36	↓18.0	0.003	3.28 ± 0.26	4.25 ± 0.41	↓29.8	0.001

## Data Availability

The data presented in this study are available on request from the corresponding author.
